# Dasatinib-loaded albumin nanoparticles possess diminished endothelial cell barrier disruption and retain potent anti-leukemia cell activity

**DOI:** 10.18632/oncotarget.10435

**Published:** 2016-07-06

**Authors:** Chunling Dong, Bo Li, Zhenyu Li, Sreerama Shetty, Jian Fu

**Affiliations:** ^1^ Department of Respiratory Medicine, Second Hospital, Jilin University, Changchun, Jilin, P.R. China; ^2^ Department of Human Anatomy, College of Basic Medical Sciences, Jilin University, Changchun, Jilin, P.R. China; ^3^ Division of Cardiovascular Medicine, College of Medicine, University of Kentucky, Lexington, KY, USA; ^4^ Center for Biomedical Research, University of Texas Health Science Center at Tyler, Tyler, TX, USA; ^5^ Center for Research on Environmental Disease, College of Medicine, University of Kentucky, Lexington, KY, USA; ^6^ Department of Toxicology and Cancer Biology, College of Medicine, University of Kentucky, Lexington, KY, USA

**Keywords:** tyrosine kinase, endothelial barrier, leukemia, drug carrier, nanoparticles

## Abstract

Dasatinib (DAS), a second-generation tyrosine kinase inhibitor, is highly effective in treating chronic myeloid leukemia and Philadelphia chromosome-positive acute lymphoblastic leukemia. However, its clinical use is limited due to serious adverse effects. DAS can disrupt endothelial barrier integrity and increase endothelial permeability which may cause peripheral edema and pleural effusion. Albumin nanoparticles (NPs) as a drug carrier may serve as a useful tool for cell-selective drug delivery to reduce DAS-induced endothelial hyperpermeability and maintain endothelial barrier integrity. In this study, we reported that DAS-loaded NPs exhibited potent anti-leukemia efficacy as DAS alone. Importantly, albumin NPs as a drug carrier markedly reduced DAS-induced endothelial hyperpermeability by restraining the inhibition of Lyn kinase signaling pathway in endothelial cells. Therefore, albumin NPs could be a potential tool to improve anti-leukemia efficacy of DAS through its cell-selective effects.

## INTRODUCTION

Chronic myeloid leukemia (CML) and Philadelphia chromosome-positive acute lymphoblastic leukemia (ALL) are the most common types of leukemia with increasing morbidity [1–3]. Dasatinib (DAS), a small molecule tyrosine kinase inhibitor, can effectively fight against CML and ALL by inhibiting the activity of both Src and BCR-ABL tyrosine kinases in leukemia cells [4, 5]. However, DAS treatment has been reported to cause serious hematologic and non-hematologic adverse effects due to its interaction with non-disease-related processes and cells, which often leads to a dose reduction or treatment discontinuation in clinic [6]. Peripheral edema and pleural effusion are the common non-hematologic side effects occurred during DAS treatment, which is likely caused by endothelial hyperpermeability [7–9]. We reported previously that the basal activity of Lyn, a member of Src kinase family, is required to maintain endothelial barrier integrity [10]. Inhibition of Lyn kinase by DAS disrupts endothelial barrier integrity, resulting in increased endothelial permeability [10].

Nanoparticles (NPs) as a drug delivery system could enhance drug bioavailability, increase specificity, improve tissue selectivity and prolong pharmacological effect resulting in higher therapeutic efficacy [11–14]. Meanwhile, the adverse effects of drugs may be effectively eliminated by employing NPs as a drug carrier. Albumin NPs have become a promising carrier for drug delivery due to their merits in bioavailability, biocompatibility and biodegradability [15, 16]. It was reported that piceatannol-loaded albumin NPs were able to prevent vascular inflammation by targeting activated neutrophils adherent to inflamed endothelium [17]. Albumin NPs loaded with paclitaxel also exhibited an enhanced anti-tumor efficacy [18]. It was reported that albumin NPs were not internalized by the TNF-α-activated endothelium [17].

Avoiding direct interaction of DAS with Lyn kinase in endothelial cells may be a useful approach to prevent DAS-induced endothelial hyperpermeability. In this study, we investigated the possibility that the DAS-induced endothelial hyperpermeability could be prevented by employing albumin NPs as a drug carrier. Human leukemic K562 cells and human pulmonary artery endothelial cells (HPAECs) were used to investigate anti-leukemia activity of DAS and DAS-loaded NPs, and their adverse effects on endothelial barrier function. Moreover, we investigated the effects DAS-loaded NPs on Lyn-mediated cell signaling in endothelial cells, including Lyn phosphorylation, VE-cadherin localization, and focal adhesion kinase (FAK) modulation.

## RESULTS

### Characterization of albumin NPs

**Figure 1:**
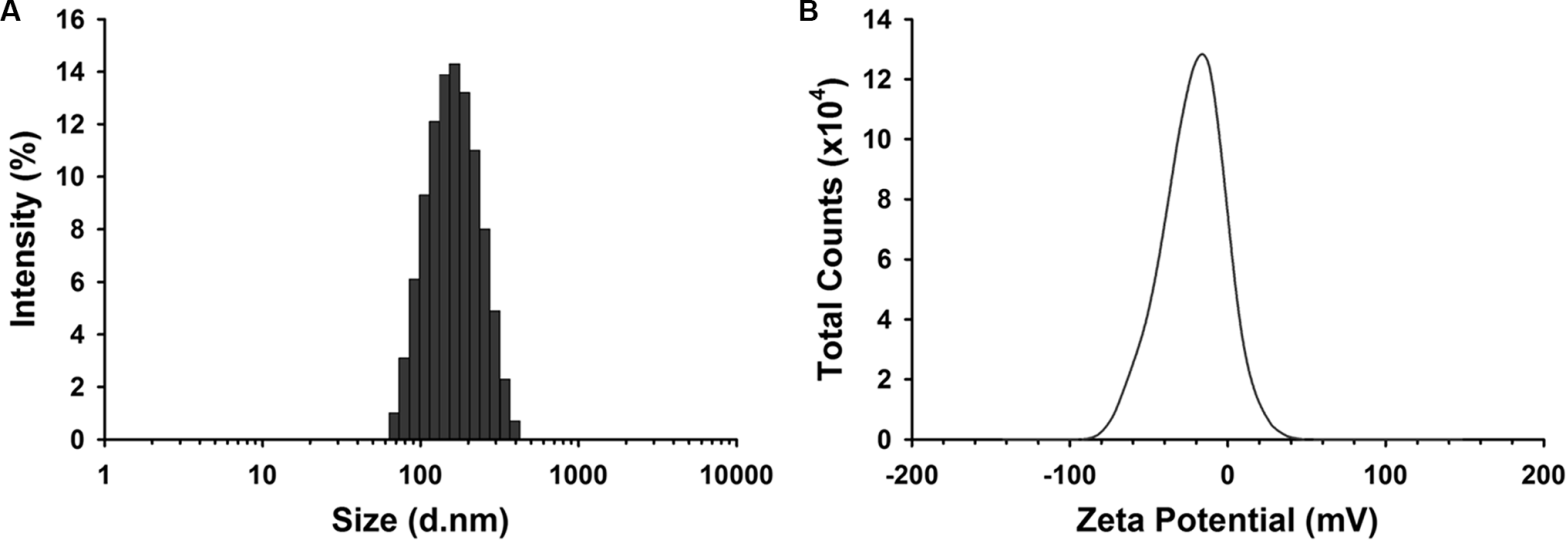
Size (A) and Zeta potential (B) distribution of DAS-loaded albumin NPs The size and Zeta potential distribution of DAS-loaded albumin NPs were measured. Data are representatives of three independent experiments.

The results of dynamic light scattering showed that albumin NPs with and without DAS loading were similar in size and polydispersity index (PDI). The average diameter of DAS-loaded albumin NPs was 148.0 ± 0.5 nm, and the PDI was 0.163 ± 0.016 (Figure 1A). The Zeta Potential of DAS-loaded albumin NPs was −19.7 ± 1.4 mV (Figure 1B). After preparing the albumin NPs, the loading yield of DAS in albumin NPs was 10.4–15.1 wt%.

### Effect of DAS and DAS-loaded NPs on leukemia cell viability

**Figure 2:**
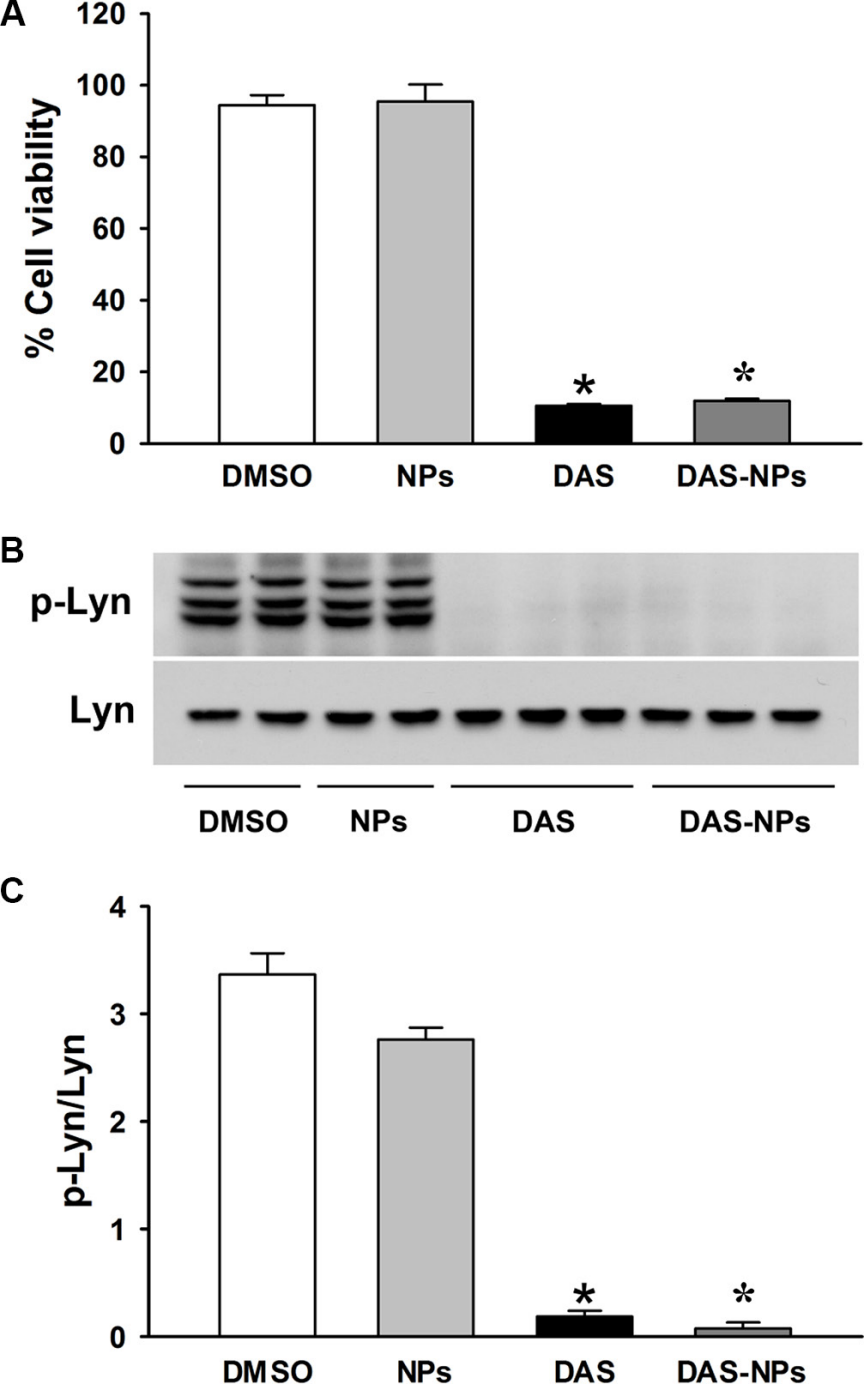
Effects of DAS and DAS-loaded NPs on K562 cell viability and modulation of Lyn kinase K562 cells were treated with DMSO, NPs, DAS (100 nM) or DAS-loaded NPs (DAS-NPs, 100 nM as DAS). (**A**) Cell viability was assessed by growth inhibition assay at 72 h after exposure. (**B**) Representative blots of phosphorylated Lyn (p-Lyn) and total Lyn (Lyn) kinases 2 h after the treatment. (**C**) Densitometry analysis is presented as relative ratios of p-Lyn to Lyn. Data were expressed as mean ± SEM. The experiments were repeated three times. *P < 0.05 versus DMSO group.

Our results showed that both DAS and DAS-loaded NPs significantly inhibited the viability of K562 cells compared to vehicle treated corresponding controls (Figure 2A). Furthermore, there was no significant difference in viability reduction between DAS and DAS-loaded NPs treated cells. These results indicate that DAS-loaded NPs possess the same anti-leukemia activity as DAS.

**Figure 3:**
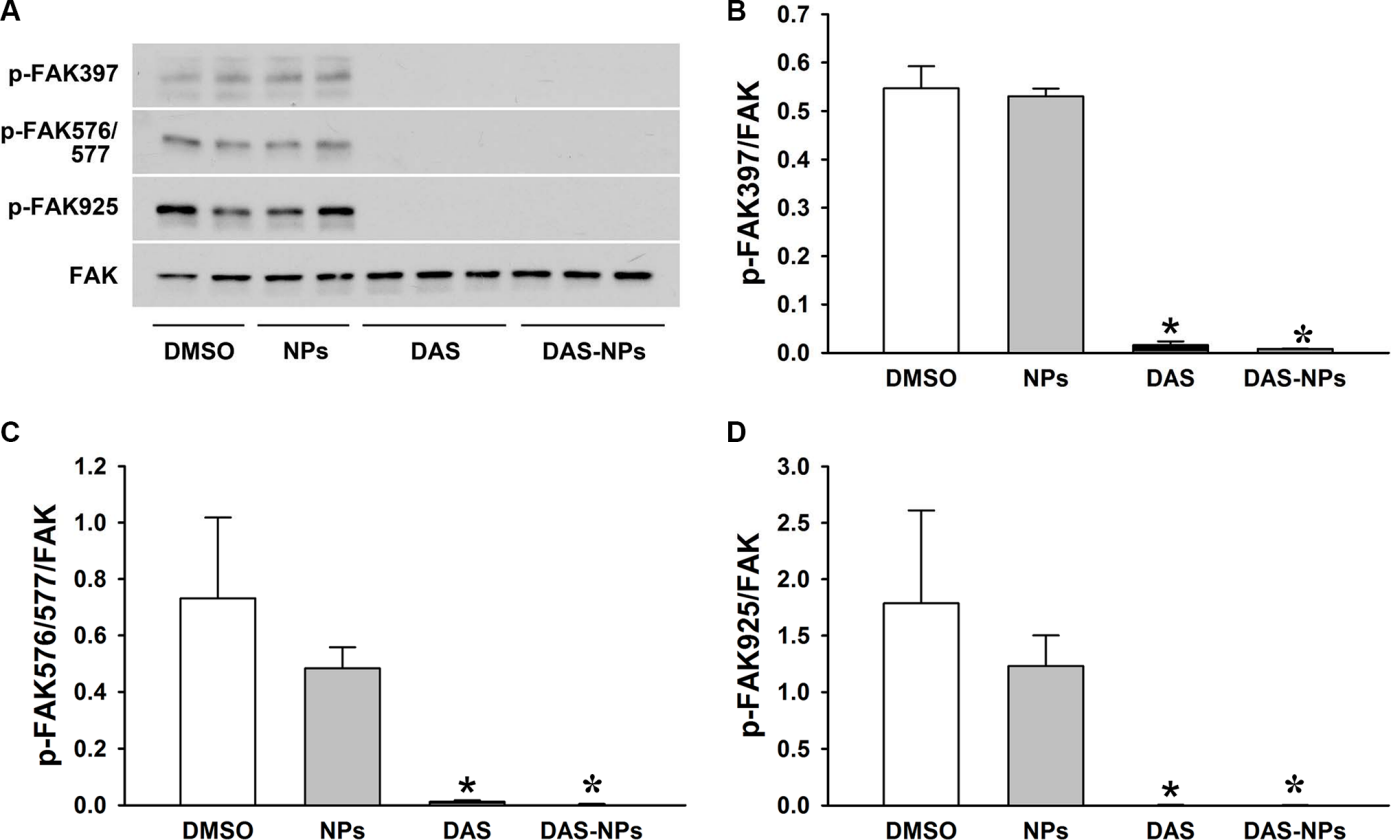
Effects of DAS and DAS-loaded NPs on FAK modulation in K562 cells K562 cells were treated with DMSO, NPs, DAS (100 nM) or DAS-loaded NPs (DAS-NPs, 100 nM as DAS) for 2 h. (**A**) Representative blots of phosphorylated FAK (p-FAK-397, p-FAK-576/577 and p-FAK-925) and total FAK (FAK). (**B**–**D**) Densitometry analysis is presented as relative ratios of p-FAK-397, p-FAK-576/577 and p-FAK-925 to FAK. Data were expressed as mean ± SEM. The experiments were repeated three times. *P < 0.05 versus DMSO group.

DAS is known to possess potent anti-leukemia activity through its inhibition of Src family kinases including Lyn [23–25]. To further assess the effects of DAS and DAS-loaded NPs on leukemia inhibition, phosphorylation levels of Lyn kinase (Figure 2B and 2C) and FAK (Figure 3) in K562 cells were evaluated 2 h after the treatment. DAS and DAS-loaded NPs significantly suppressed the phosphorylation of Lyn kinase, and the phosphorylation of FAK at residues Tyr397, Tyr576/577 and Tyr925. There were no significant differences in the phosphorylation levels of Lyn kinase and FAK in K562 cells between DAS and DAS-loaded NPs. These results indicate that the synthesized DAS-loaded NPs are as potent as DAS in leukemia inhibition.

### Effect of DAS and DAS-loaded NPs on endothelial barrier function

**Figure 4:**
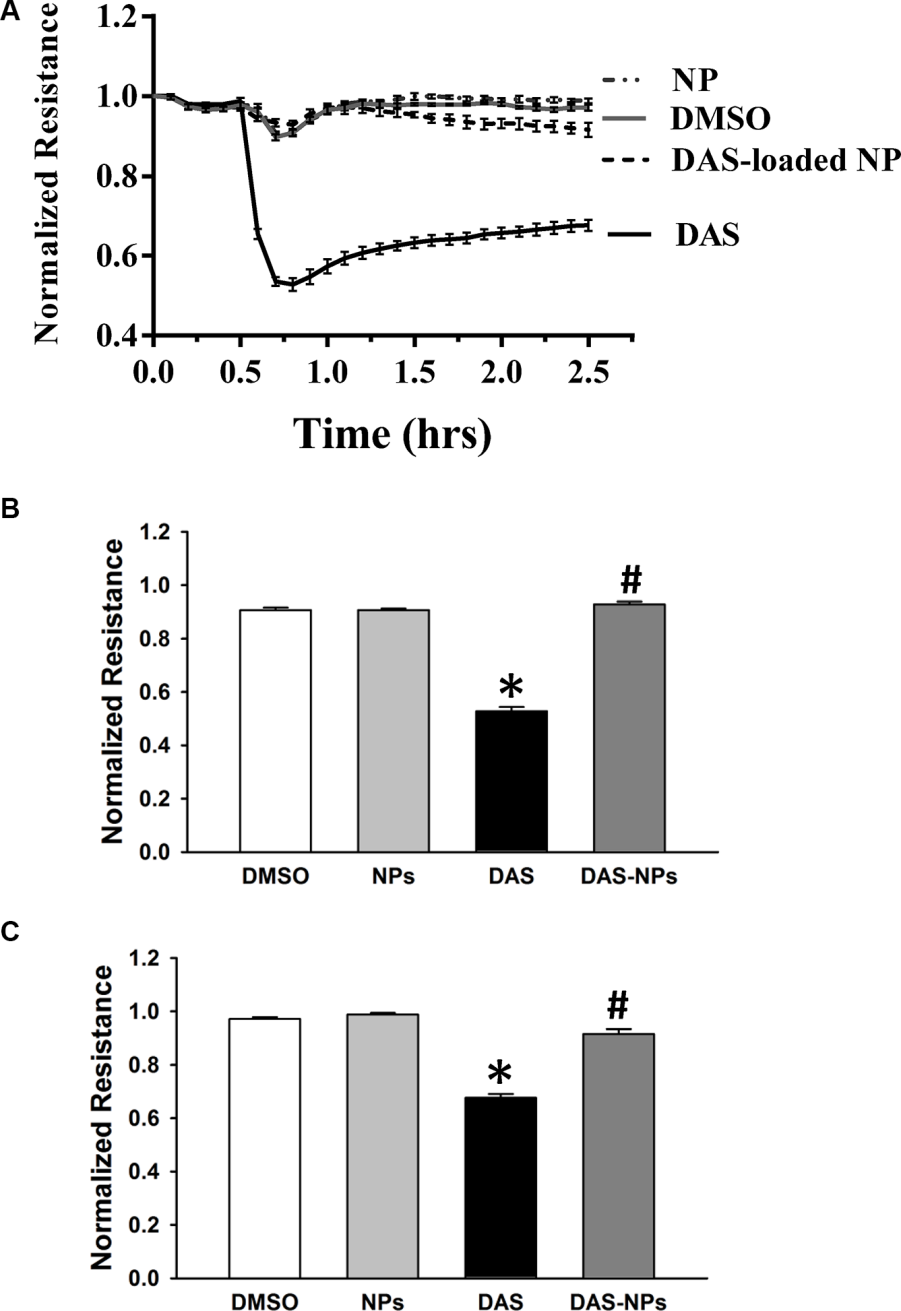
Effects of DAS and DAS-loaded NPs on TER across HPAECs HPAECs were grown to confluence on gold microelectrodes arrays, then treated with DMSO, NPs, DAS (100 nM) or DAS-loaded NPs (DAS-NPs, 100 nM as DAS). TER across HPAECs monolayers was measured. (**A**) TER dynamic changes in 2.5 h. (**B**) Representative TER values at 0.8 h after exposure. (**C**) Representative TER values at 2.5 h after exposure. Data were expressed as mean ± SEM of four independent experiments. *P < 0.05 versus DMSO group; ^#^*P* < 0.05 versus DAS group.

To assess the effect of DAS and DAS-loaded NPs on endothelial barrier function, basal TER, an assay for endothelial barrier integrity, was measured at a different time points in HPAECs. Under the treatment of DAS, basal TER began to decrease from 0.5 h, reached its lowest point at 0.8 h (Figure 4), indicating that DAS-induced disruption of endothelial barrier integrity caused the increase of endothelial permeability. However, the treatment with DAS-loaded NPs had little effect on basal TER when compared with that of DAS. Our results indicate that albumin NPs as a drug carrier diminish endothelial barrier disruption caused by DAS.

### Effect of DAS and DAS-loaded NPs on adherent junctions in endothelial cells

**Figure 5:**
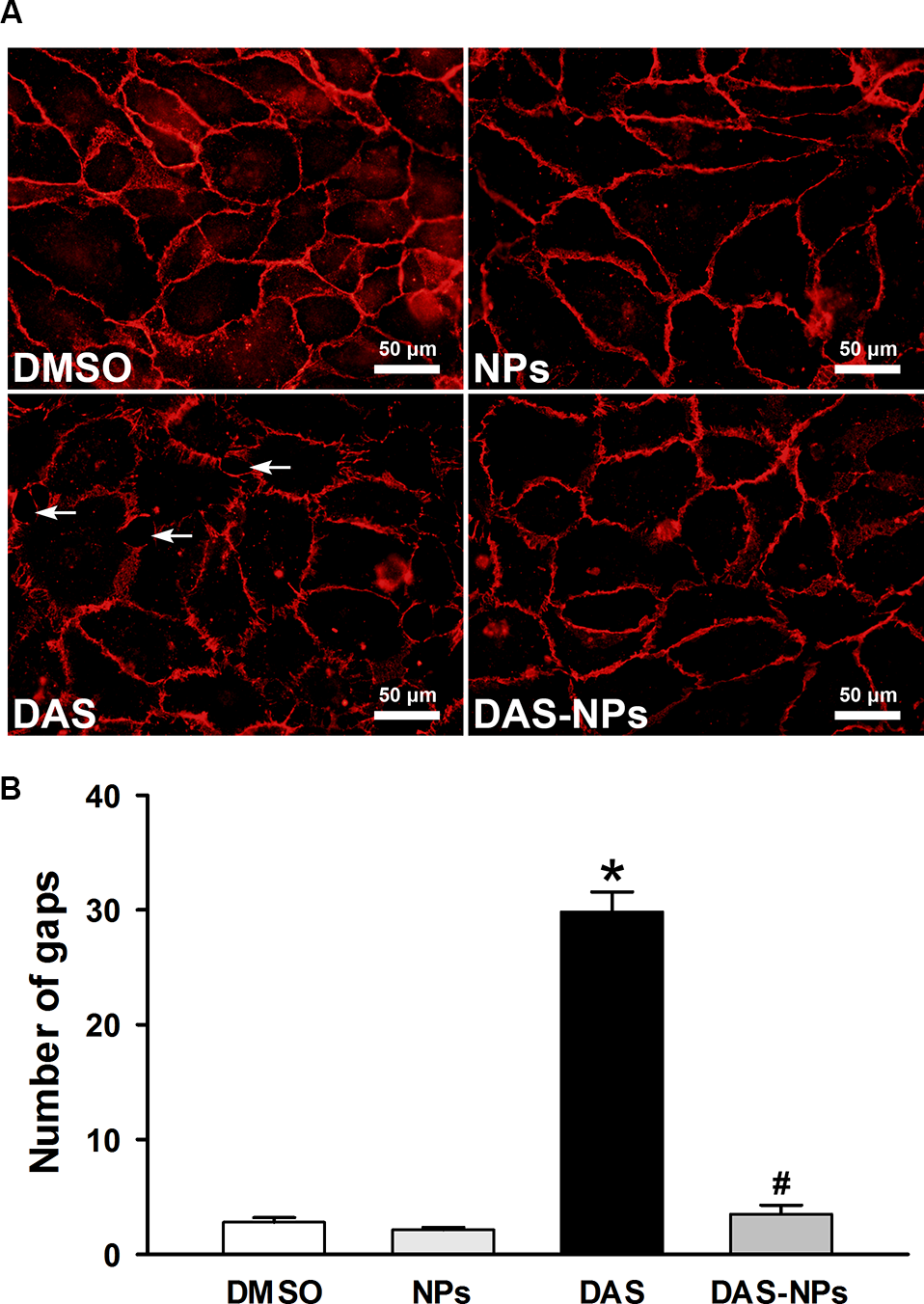
Effects of DAS and DAS-loaded NPs on adherent junctions between HPAECs HPAECs were treated with DMSO, NPs, DAS (100 nM) or DAS-loaded NPs (DAS-NPs, 100 nM as DAS) for 2 h. (**A**) Immunofluorescence staining for VE-cadherin subcellular distribution and formation of interendothelial junctional gaps (White arrow). Quantification of gaps (**B**) in 10 random images was expressed as mean ± SEM of three independent experiments. *P < 0.05 versus DMSO group; ^#^*P* < 0.05 versus DAS group.

The subcellular distribution of VE-cadherin, as a molecular marker of vascular endothelial adherent junctions, was examined in HPAECs monolayers to visualize the effect of DAS and DAS-loaded NPs on the integrity of endothelial junctions. DAS caused the distinct discontinuities in VE-cadherin distribution between the cells and formation of numerous interendothelial junctional gaps (White arrow) at 2 h, indicating the DAS disrupted adherent junction integrity (Figure 5). Interesting, DAS-loaded NPs had little effect on VE-cadherin distribution. The number of interendothelial junctional gaps was significantly decreased under the treatment of DAS-loaded NPs when compared with that of DAS-treated cells. The results further demonstrate that albumin NPs as a drug carrier retain endothelial barrier integrity.

### Effect of DAS and DAS-loaded NPs on Lyn signaling in endothelial cells

**Figure 6:**
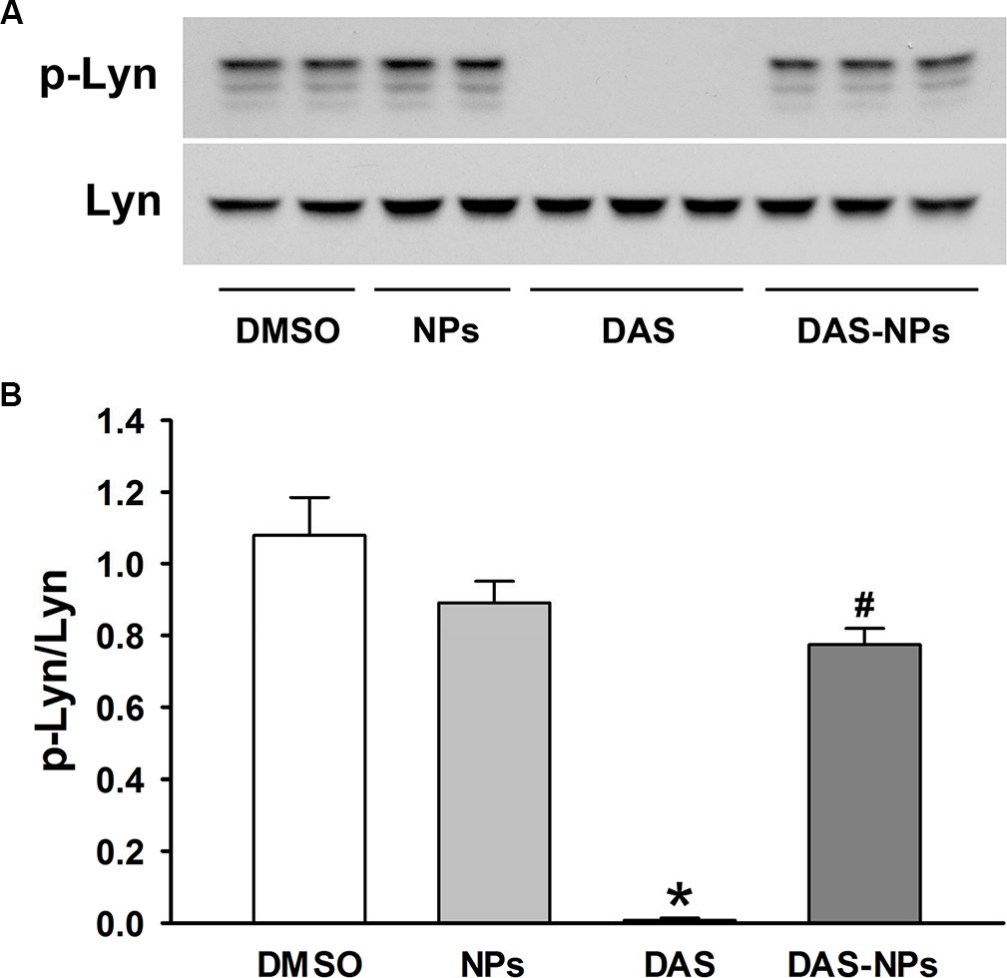
Effects of DAS and DAS-loaded NPs on Lyn signaling in HPAECs HPAECs were treated with DMSO, NPs, DAS (100 nM) or DAS-loaded NPs (DAS-NPs, 100 nM as DAS) for 2 h. (**A**) Representative blots showing phosphorylated Lyn (p-Lyn) and total Lyn (Lyn) kinases. (**B**) Densitometry analysis is presented as relative ratios of p-Lyn to Lyn. Data were expressed as mean ± SEM. The experiments were repeated three times.*P < 0.05 versus DMSO group; ^#^*P* < 0.05 versus DAS group.

We then examined the effect of DAS and DAS-loaded NPs on Lyn kinase signaling in endothelial cells. DAS treatment significantly decreased the phosphorylation of Lyn kinase in HPAECs (Figure 6), while the treatment with DAS-loaded NPs had little effects on Lyn phosphorylation. These results indicated that DAS-induced Lyn kinase inhibition was reduced by employing albumin NPs as drug carrier.

**Figure 7:**
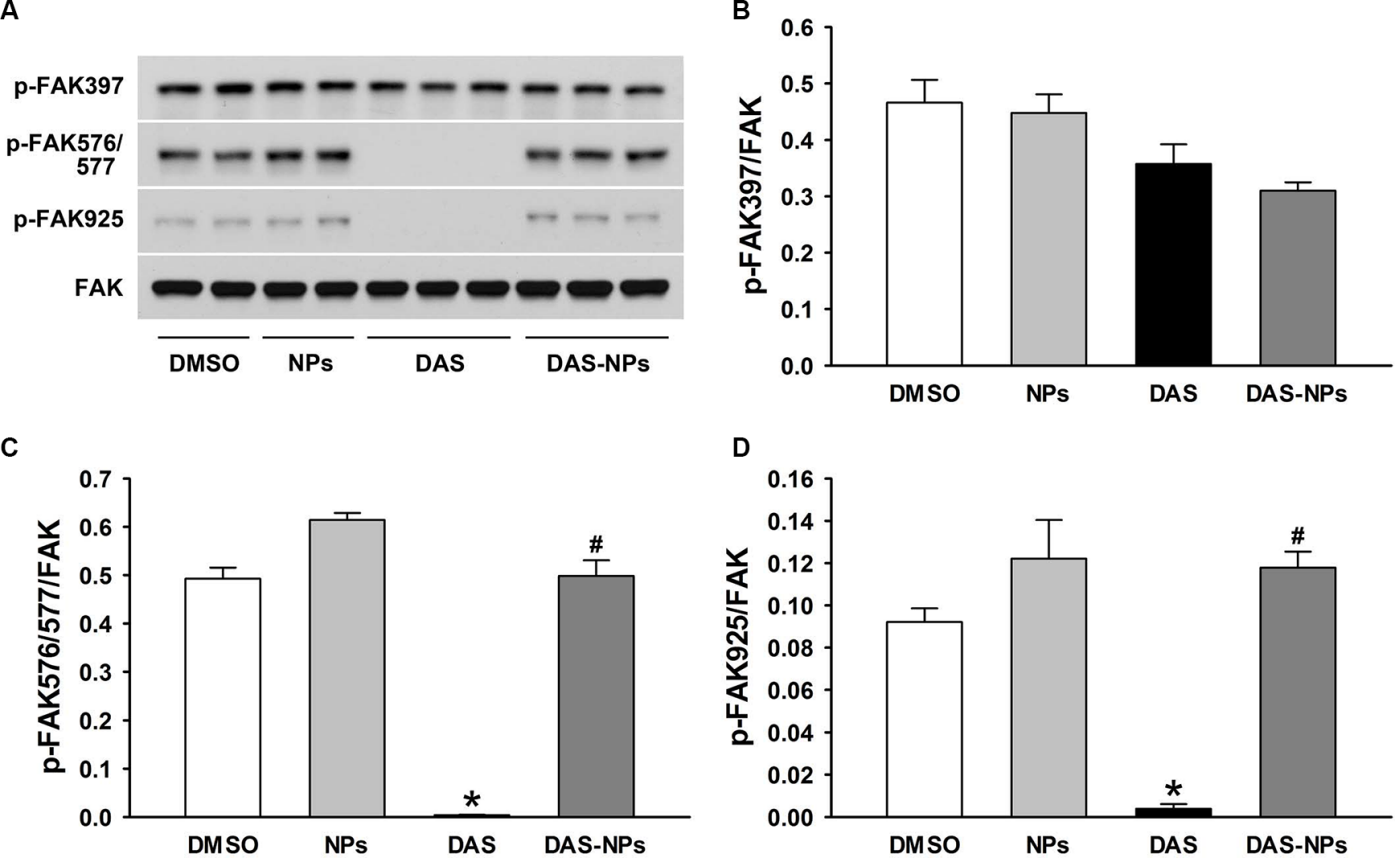
Effects of DAS and DAS-loaded NPs on modulation of FAK in HPAECs HPAECs were treated with DMSO, NPs, DAS (100 nM) or DAS-loaded NPs (DAS-NPs, 100 nM as DAS) for 2 h. (**A**) Representative blots showing phosphorylated (p-FAK-397, p-FAK-576/577 and p-FAK-925) and total FAK. (**B**–**D**) Densitometry analysis is presented as relative ratios of p-FAK-397, p-FAK-576/577 and p-FAK-925 to FAK. Data were expressed as mean ± SEM. The experiments were repeated three times. *P < 0.05 versus DMSO group; ^#^*P* < 0.05 versus DAS group.

We previously reported that Lyn kinase-mediated regulation of FAK activity plays a pivotal role in maintaining endothelial barrier function [10]. To confirm the involvement of FAK in DAS-induced endothelial hyperpermeability, FAK expression was evaluated after the treatment with DAS and DAS-loaded NPs. In our study, DAS treatment significantly decreased the phosphorylation of FAK at residues Tyr576/577 and Tyr925 without significantly affecting phosphorylation of FAK at residue Tyr397 (Figure 7). However, DAS-loaded NPs had little effects on FAK phosphorylation at residues Tyr576/577 and Tyr925 in HPAECs. Our results indicate that albumin NPs as a drug carrier reduce the inhibition of FAK-medicated signaling by DAS in endothelial cells.

### Uptake of albumin NPs by leukemia cells and endothelial cells

**Figure 8:**
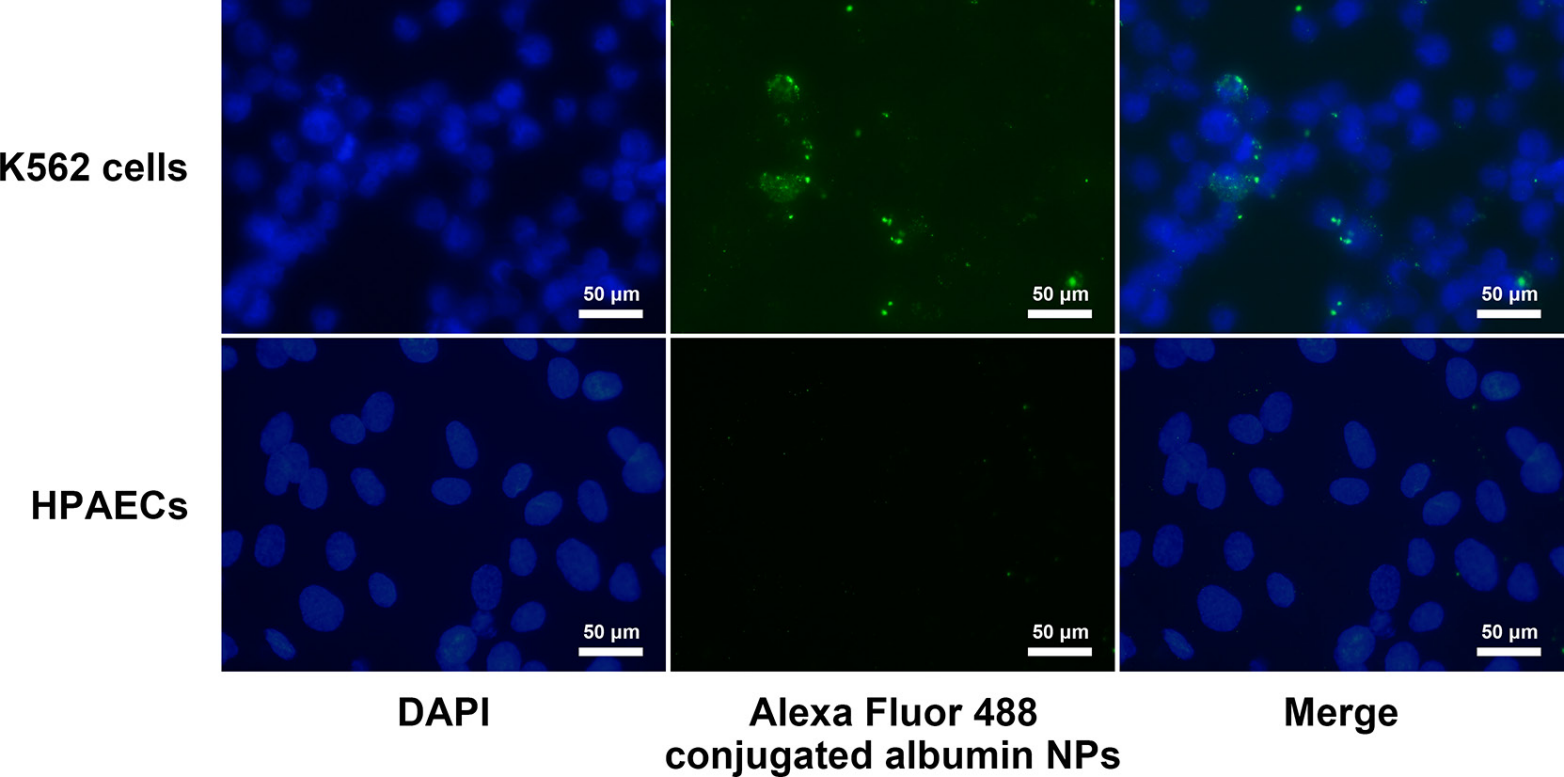
Uptake of albumin NPs by K562 and HPAECs K562 cells and HPAECs were incubated with Alexa Fluor 488-conjugated albumin NPs for 2 h. Cells were then washed, fixed, and visualized by fluorescence microscopy. Images are representatives of three independent experiments.

To address the cell-selective effects of DAS NPs, we examined the uptake of Alexa Fluor 488 conjugated albumin NPs by K562 cells and HPAECs. Our results showed that Alexa Fluor 488-conjugated albumin NPs were efficiently internalized by K562 cells, while little green fluorescence was observed in HPAECs, indicating that albumin NPs failed to enter HPAECs (Figure 8).

## DISCUSSION

Adverse effects of anti-cancer drugs often lead to a dose reduction or discontinuation of treatments in clinical settings. NPs as a drug delivery system have been reported to serve as an effective approach to enhance the drug efficacy and reduce adverse effects [13]. Albumin NPs have been reported to serve as a promising drug carrier for treating inflammatory diseases and cancer [17, 18, 26, 27]. In this study, we successfully synthesized albumin NPs loaded with DAS. Albumin NPs were prepared according to a modified desolvation method, which could assure DAS encapsulated in the NPs [19, 28]. DAS-loaded NPs possessed potent anti-leukemic activity as DAS. Importantly, albumin NPs as a drug carrier markedly reduced DAS-induced endothelial hyperpermeability by restraining the inhibition of Lyn-FAK signaling pathway.

In our study, the anti-leukemia activity of DAS and DAS-loaded NPs was assessed in K562 leukemia cells. Our results showed that DAS-loaded NPs possessed potent anti-leukemia activity as DAS. The key of NPs as a drug carrier lies in the successful uptake of drug-loaded NPs by target cells. It was reported that albumin NPs made from denatured albumin could be internalized by activated neutrophils [17]. DAS-loaded NPs may be internalized by K562 leukemia cells through FcγR [29]. DAS released from albumin NPs can then bind to Src and BCR-ABL tyrosine kinases and inhibit their autophosphorylation along with their downstream targets leading to blockade of the oncogenic activities of leukemia cells [30, 31]. Future studies using animal models of leukemia are needed to confirm our *in vitro* data. The *in vivo* studies could provide important information on therapeutic potential of DAS-loaded NPs.

We previously reported that inhibition of Lyn kinase disrupts endothelial barrier integrity [10]. Lyn, unlike other members of the Src kinase family including c-Src, Fyn, and Yes, strengthens endothelial barrier integrity through phosphorylation of FAK at residues Tyr576/577 and Tyr925. FAK, a well-known Src family kinase (SFK) substrate, plays a critical role in maintaining endothelial barrier function [32, 33]. Down-regulation of FAK was reported to cause disruption of adherent junctions at endothelial borders resulting in formation of intercellular gaps [10], indicating that FAK plays an important role in re-establishing adherent junctions in endothelial cells. In addition, FAK is a major determinant of vascular permeability by regulating focal adhesion formation and turnover [34].

In this study, we demonstrated that DAS treatment disrupts endothelial barrier integrity in HPAECs by inhibiting Lyn-FAK signaling, which is characterized by the formation of intercellular gaps between adjacent endothelial cells and reduced TER. The phosphorylation of endothelial Lyn and FAK was inhibited by DAS in endothelial cells. FAK phosphorylation at residues Tyr576/577 and Tyr925, not Tyr397, was also inhibited by DAS, which is consistent with our previous report on Lyn regulation of endothelial barrier function [10]. The subsequent vascular leakage due to endothelial hyperpermeability could lead to edema formation.

Albumin NPs as a promising drug carrier could increase specificity and enhance therapeutic efficacy [17, 18]. Fluorescently tagged albumin NPs failed to be internalized by endothelial cells [17]. In our study, Albumin NPs entered leukemia cells efficiently, but exhibited little uptake by endothelial cells. The failed endocytosis of DAS-loaded NPs by HPAECs may be due to the lack of Fcγ receptors on endothelial cells [35]. Thus, the interaction of DAS with Lyn kinase in endothelial cells was prevented when DAS was loaded in albumin NPs. DAS-loaded NPs was not able to inhibit the phosphorylation of endothelial Lyn and FAK and had little effect on endothelial permeability. The adverse effects of DAS on endothelial barrier integrity were effectively eliminated by employing albumin NPs as a drug carrier. The albumin nanoparticles are designed by us to deliver DAS selectively to leukocytes with less toxicity to endothelial cells. It was also reported previously by others that piceatannol-loaded albumin NPs were able to inhibit vascular inflammation by targeting activated neutrophils adherent to endothelial cells [17]. The albumin NPs are not designed to be more selective to leukemia cells than to normal leukocytes yet. Hopefully, in the future we could also develop albumin NPs to selectively target leukemia cells and with less toxicity to normal leukocytes.

In summary, we have demonstrated that DAS-loaded albumin NPs exhibited potent anti-leukemia activity as DAS. The disruption of endothelial barrier integrity by DAS was markedly reduced by employing albumin NPs as drug carrier. DAS-loaded NPs may offer a new treatment option for patients with CML or Ph-positive ALL who are unable to tolerate DAS-induced edema and other fluid retention. Albumin NPs as a drug delivery system could improve the anti-leukemia efficacy of DAS through its cell-selective effects. DAS-loaded NPs are potential therapy for leukemia with better safety profile and anti-leukemia efficacy. This study could help us improve the formulation of drugs with similar adverse effects as DAS. Further studies are warranted to assess the potential application of DAS-loaded albumin NPs in clinic.

## MATERIALS AND METHODS

### Materials

Human serum albumin (HSA, purity ≥ 99%) and 8% glutaraldehyde solution were purchased from Sigma–Aldrich Company (St. Louis, MO, USA). DAS (Purity: > 99%) was purchased from Cell Signaling Technology (Beverly, MA, USA). All other chemicals were purchased from Fisher Scientific (Pittsburgh, PA, USA). Rabbit polyclonal antibodies against phosphorylated FAK at residues Tyr397, 576/577, and 925, phosphorylated Lyn and total FAK, and a rabbit monoclonal antibody against total Lyn were obtained from Cell Signaling Technology (Beverly, MA, USA). A goat polyclonal antibody against VE-Cadherin was obtained from Santa Cruz Biotechnology (Santa Cruz, CA, USA).

### Synthesis and characterization of albumin NPs

Albumin NPs were prepared according to a modified desolvation method [19]. Briefly, HSA was dissolved at a concentration of 20 mg/ml in 10 mM sodium chloride solution and the pH of the solution was titrated to 8. The resulting solutions were filtered through a 0.22 μm filtration unit (EMD Millipore Corporation, Billerica, MA, USA). To make DAS-loaded albumin NPs, HSA solution (1 ml) was incubated with 1 mg of DAS dissolved in DMSO for 1 h. Then, HSA solution (1 ml) with or without DAS co-incubation was transformed into NPs by the continuous addition of 4 ml of the desolvating agent methanol under stirring (550 rpm) at room temperature. The methanol addition was performed by a tubing pump (FH10, Thermo Fisher Scientific, Barrington, IL, USA) with a defined rate of 1 ml/min. After the desolvation process 24 μl of 8% glutaraldehyde in water were added to induce particle crosslinking. The crosslinking process was performed under stirring (200 rpm) of the suspension over a time period of 24 h at room temperature. The nanoparticle suspension was centrifuged at 14,000 rpm for 30 min at 4°C. The nanoparticle pellet was re-suspended in phosphate-buffered saline (pH 7.4).

The size and Zeta potential distribution of DAS-loaded albumin NPs were measured using a Malvern Zetasizer Nano ZS Instrument (Malvern Instruments Ltd., Malvern, Worcestershire, UK). To evaluate the loading efficiency of DAS in albumin NPs, the supernatant (containing unbound DAS) after centrifugation of a suspension of DAS-loaded albumin NPs was collected and centrifuged using 10 kDa Microcon (EMD Millipore Corporation, Billerica, MA, USA) to separate unbound DAS (Molecular Weight: 488.01 Da) from free HSA (Molecular Weight: 66478 Da) molecules. DAS molecules in filtrate were quantified by measuring the absorbance at 282 nm. The loading yield of DAS in albumin NPs was calculated with the following equation: loading yield (%) = (drug used–unloaded drug)/drug used.

### Cell culture

Primary HPAECs were purchased from Lonza Walkersville Inc. (Conshohocken, PA, USA). These cells were cultured with endothelial growth medium-2 (EGM-2, Lonza Walkersville Inc.) and supplemented with 10% fetal bovine serum (FBS). Human leukemic K562 cells (kindly provided by Dr. Ying Liang, University of Kentucky) were cultured with Iscove's Modified Dulbecco's Medium (IMDM, Thermo Scientific, Logan, UT, USA) supplemented with 10% heat-inactivated fetal bovine serum (FBS), penicillin and streptomycin (1%) and cultured at 37°C in a humidified incubator with 5% CO_2_.

### Growth inhibition assay

CellTiter 96 Aqueous Non-Radioactive cell proliferation Assay Kit (Promega Corp., Madison, Wisconsin, USA) was used for growth inhibition assay as previously described [20]. 10,000 K562 cells were plated in 96-well flat-bottomed plates and cultured for 24 h. Cells were exposed to DMSO, NPs, DAS (100 nM) or DAS-loaded NPs (100 nM as DAS) in IMDM with 10% FBS, for an additional 72 h. 20 μl MTS/PMS solution was added into each well containing 100 μl of the culture medium. Then, the cells were incubated for 3 h at 37°C before measurement of absorbance at 490 nm with an Epoch microplate spectrophotometer (BioTek Instruments Inc., Winooski, VT, USA). Absorbance values changes were normalized to that for untreated cells.

### Measurement of transendothelial electrical resistance

Real-time changes in transendothelial electrical resistance (TER) across endothelial monolayers were measured to evaluate the endothelial barrier function as previously described [21]. Briefly, HPAECs were seeded onto gold microelectrode arrays (Applied Biophysics Inc., Troy, NY, USA) and cultured to confluence. Cells were exposed to DMSO, NPs, DAS (100 nM) or DAS-loaded NPs (100 nM as DAS). After exposure, impedance across the cells was measured for 2.5 h, and the TER changes were normalized to its initial value.

### Immunofluorescence staining

To assess the effect of DAS and DAS-loaded NPs on adherent junctions between endothelial cells, HPAECs cultured onto 0.2% gelatin-coated coverslips were incubated with DMSO, NPs, DAS (100 nM) or DAS-loaded NPs (100 nM as DAS) for 2 h. Cells were fixed with 4% paraformaldehyde for 20 minutes, followed by permeabilization with 0.1% Triton ×-100. Thereafter, cells were incubated with primary antibody against endogenous VE-cadherin for 1 h, followed by incubation with Alexa-Fluor 594 labeled donkey anti-goat secondary antibody, using Hanks balanced salt solution containing 1% bovine serum albumin as a blocking buffer. Images were acquired using an Olympus BX43 microscope (Olympus Corporation, Tokyo, Japan).

### Cellular uptake of albumin NPs

Alexa Fluor 488 Protein Labeling Kit (Molecular Probes/Invitrogen, Eugene, OR, USA) was used to label albumin NPs. In brief, 0.5 ml of 2 mg/ml albumin NPs suspended in phosphate-buffered saline was mixed with 50 μl of 1 M sodium bicarbonate solution. Then, the mixture was transferred to the provided vial of Alexa Fluor 488 reactive dye and reacted for 1 h at room temperature. After reaction, the Alexa Fluor 488 conjugated albumin NPs were purified by three cycles of centrifugation (14,000 rpm for 30 min at 4°C) and re-suspended in phosphate-buffered saline. K562 cells were seeded onto 24-well culture plates, with approximately 2.5 × 10^5^ cells in 1 ml of medium. HPAECs were cultured onto 0.2% gelatin-coated coverslips placed in 24-well culture plates. K562 cells and HPAECs were cultured for 24 h, then exposed to the Alexa Fluor 488-conjugated albumin NPs (40 μg) for 2 h. The cells were washed three times with phosphate-buffered saline and fixed with −20°C 100% methanol for 10 minutes. After fixation, the cells were washed three times with phosphate-buffered saline. The coverslips with HPAECs were put on the slides coated with DAPI for nuclear staining. K562 cells were smeared on the surface of the slides and stained with DAPI. The uptake of albumin NPs by K562 cells and HPAECs was visualized using an Olympus BX43 microscope (Olympus Corporation, Tokyo, Japan).

### Immunoblotting assay

*Approximately* 4 × 10^5^ HPAECs or K562 cells were seeded into 6-well plates in serum-containing medium. After culture for 24 h, cells were exposed to DMSO, NPs, DAS (100 nM) or DAS-loaded NPs (100 nM as DAS) for 2 h. The cells were lysed for protein extraction using cell lysis buffer with protease inhibitor and phosphatase inhibitor (Cell Signaling Technology, Beverly, MA, USA). The total protein concentration was measured by BCA Protein Assay Kit (Pierce Biotechnology, Rockford, IL, USA). Isolated proteins (30 μg/Lane) were separated by SDS-PAGE and transferred to a polyvinylidene fluoride (PVDF) membrane for immunoblotting assay as described previously [22].

### Statistical analyses

Statistics were performed using GraphPad Prism 6 software (GraphPad Prism Software Inc., San Diego, CA, USA). Data were expressed as mean ± SEM. A one-way analysis of variance followed by Turkey's test (multiple groups) was performed to determine the statistical significance (*P* < 0.05) between the indicated groups.
